# Disentangling SARS-CoV-2 Sustained Viremia Cases: Evolution, Persistence and Reinfection

**DOI:** 10.3390/v18030393

**Published:** 2026-03-21

**Authors:** Brunna M. Alves, Filipe R. R. Moreira, Marianne M. Garrido, Pedro S. de Carvalho, Élida M. de Oliveira, Caroline C. de Sá, James Arthos, Claudia Cicala, João P. B. Viola, Livia R. Goes, Juliana D. Siqueira, Marcelo A. Soares

**Affiliations:** 1 Programa de Genética e Virologia Tumoral, Instituto Nacional de Câncer, Rio de Janeiro 20231-050, Brazil; brunna_alves@yahoo.com.br (B.M.A.); pedro42sw@gmail.com (P.S.d.C.); mendeselida16@gmail.com (É.M.d.O.); cdsacaroline@gmail.com (C.C.d.S.); liviargoes@gmail.com (L.R.G.); sidoju.juliana@gmail.com (J.D.S.); 2 Departamento de Genética, Universidade Federal do Rio de Janeiro, Rio de Janeiro 21941-901, Brazil; filiperomero2@gmail.com; 3 Comitê de Controle de Infecção Hospitalar, Instituto Nacional de Câncer, Rio de Janeiro 20230-130, Brazil; marianne.monteiro@inca.gov.br; 4 Laboratory of Immunoregulation, National Institute of Allergy and Infectious Diseases, Bethesda, MD 20892, USA; jarthos@niaid.nih.gov (J.A.); ccicala@niaid.nih.gov (C.C.); 5 Programa de Imunologia e Biologia Tumoral, Instituto Nacional de Câncer, Rio de Janeiro 20231-050, Brazil; jpviola@inca.gov.br; 6 Departamento de Genética e Biologia Molecular, Universidade Federal do Estado do Rio de Janeiro, Rio de Janeiro 20211-010, Brazil

**Keywords:** SARS-CoV-2, COVID-19, cancer, intrahost dynamics, persistence

## Abstract

Based on the follow-up of patients who recovered from severe acute respiratory syndrome coronavirus 2 (SARS-CoV-2) infection, several reports of people who re-tested positive have been described. This may result from viral reactivation, true reinfection, superinfection, or an initial infection by more than one virus (multiple infection). These scenarios can only be correctly distinguished through viral quasispecies analysis. Herein, 26 cancer patients under extended follow-up for SARS-CoV-2 infection were submitted to multiple longitudinal analyses through nucleic acid isolation, PCR amplification and high-throughput sequencing. SARS-CoV-2 classification and the definition of cases as persistent or repeated infections were based on phylogenetic reconstruction. Supported by their viral complete genomes and intrahost quasispecies over time, the different scenarios were identified. Nine confirmed and 12 plausible persistence cases were identified. Virus evolution dynamics in the intrahost population from patients with persistent infection was shown for the first time. Regarding reinfection, three confirmed and two plausible cases were identified, including one case of multiple infection. Altogether, this is the first study that analyzes the plethora of SARS-CoV-2 within-host minor variants and describes reinfections, multiple infections and viral evolution across time in cancer patients, contributing to the understanding of SARS-CoV-2’s within-host population dynamics in the natural history of COVID-19.

## 1. Introduction

Since the 2019 coronavirus disease (COVID-19) description by the Chinese medical community [1], more than 778 million infections by the etiological agent of the disease, the severe acute respiratory syndrome coronavirus 2 (SARS-CoV-2), have been reported to the World Health Organization (https://covid19.who.int/ accessed on 20 December 2025) until December 2025. SARS-CoV-2, more recently named *Betacoronavirus pandemicum* by the International Committee of Taxonomy of Viruses (ICTV) [2], belongs to the genus *Betacoronavirus* of the *Coronaviridae* family. Persistent SARS-CoV-2 infections provide a prolonged environment where the virus is exposed to host immune responses and other selective pressures. This extended exposure allows for the accumulation of adaptive intrahost mutations over time [3]. In this sense, the follow-up of patients who were previously diagnosed by a positive test and recovered from the disease and have a new positive test, with or without recrudescence of COVID-19 symptoms, has become increasingly common in the literature [4,5,6,7,8,9,10].

In a well-documented case reported in late August 2021, To and colleagues described the first apparent case of reinfection of a 33 years old man from Hong Kong who tested PCR-positive for the virus after 142 days of the first symptomatic episode of infection, with two negative PCR tests within that period, during his recovery and hospital discharge. Viral reinfection was confirmed by whole-genome analysis and showed that strains from both timepoints belong to different phylogenetic clades [3]. Because a SARS-CoV-2 IgG assay was performed after five days of hospitalization in the first episode (which was positive) and one day after hospitalization in the second episode (which was negative), the authors concluded that a waning antibody response was likely accountable for re-sensitization of the subject, which allowed reinfection to occur [4].

Multiple theoretical scenarios can explain a sequence of positive–negative–positive RT-PCR results for the presence of SARS-CoV-2 in consecutive samples of an infected individual. Exclusion of the occurrence of false positives or negatives due to technical issues leaves three major hypotheses. The first one is viral reactivation, in which a unique virus entity causes a first clinical episode during viral replication (and a positive RT-PCR test), but then is somehow controlled, yet not completely eradicated by immune responses. A period of apparent viral latency follows (RT-PCR-negative), but then viral replication is again triggered by an unknown factor (likely immune-related), and viremia is again documented (RT-PCR-positive). A second possibility is a true reinfection or a superinfection, where two distinct viral strains (or variants) are involved. The first virus causes a first episode (RT-PCR positivity), followed by virus eradication or latency (RT-PCR-negative), and after some time, a second virus infects the subject, triggering the second RT-PCR-positive signal. In this scenario, waning immune responses or infection by an immune-escape variant may occur. The third possibility is an initial infection by two or more viruses simultaneously (multiple or coinfection), in which one variant outgrows the other in the viral population (RT-PCR-positive) or is controlled by the host immune responses (RT-PCR-negative), but the second variant then outgrows the first due to waning responses or through immune-escape variations. While SARS-CoV-2 genome profiling of the consensus sequences derived from the two clinical episodes distinguishes between the first and second scenario depicted above, only the analysis of the viral quasispecies circulating in the infected individual, including the determination of minor variants, allows for discrimination between the second and the third scenarios. Our group reported a case of coinfection in a cancer patient falling into that latter category, supported by the presence of single-nucleotide variations (SNVs) that characterized the second virus in the intrahost quasispecies of the first sample [11].

Herein, we describe multiple longitudinal analyses of 26 different cases of extended SARS-CoV-2 infection in cancer patients. By assessing their complete viral consensus sequence genomes, as well as their intrahost quasispecies variations over time, we were able to support the occurrence of all infection scenarios described above. Moreover, we reported the viral evolution in patients with persistent infection, with emergence and fixation of SNVs in the intrahost viral quasispecies population.

## 2. Materials and Methods

### 2.1. Study Subjects

Twenty-eight cancer patients followed at the Brazilian National Cancer Institute (INCA), Rio de Janeiro, Brazil, with more than one SARS-CoV-2-positive test within a six-month interval were included in this longitudinal study. SARS-CoV-2 tests were performed in naso/oropharyngeal swabs using real-time reverse-transcription polymerase chain reaction (RT-qPCR) following the U.S. Centers for Disease Control and Prevention (CDC) protocol [12]. SARS-CoV-2 infections were detected in early pandemics (from April to July 2020) in all cases. This study was approved by the Brazilian National Committee for Ethics in Research (CONEP) under the reference protocol CAAE 30608220.8.0000.5274. All participants provided written informed consent agreeing to be enrolled in the study, unless they were unable to sign due to several clinical conditions. In those cases, the Institutional Review Board waived the informed consent signing.

### 2.2. SARS-CoV-2 Near-Full-Length Genome Amplification and Sequencing

All methods regarding SARS-CoV-2 nucleic acid isolation, amplification and sequencing were previously described in detail [13]. Briefly, positive SARS-CoV-2 samples were subjected to viral RNA/DNA extraction with the QIAamp MinElute Virus Spin Kit (QIAGEN, Chatsworth, CA, USA). cDNA synthesis was carried out in duplicate for each sample with the SuperScript™ III First-Strand Synthesis System (Thermo Fisher Scientific, Waltham, MA, USA). Multiplex PCR reactions were conducted to amplify the SARS-CoV-2 complete genome with Platinum Taq DNA Polymerase High Fidelity (Thermo Fisher Scientific) following the SARS-CoV-2 V.3 primers protocol developed by the ARTIC network [14]. For samples for which this amplification was not successful, a semi-nested PCR was designed with the same ARTIC network V.3 primers. Both the first and second PCR rounds were carried out at the same conditions as described in the ARTIC network amplification protocol, except for the first round, which contained only part of the multiplex primers (listed on Supplementary Table S1). After purification of positive PCR products using the ReliaPrep™ DNA Clean-Up (Promega Corporation, Madison, WI, USA) and Concentration System (Promega, Madison, WI, USA), genomic libraries were constructed with Nextera XT DNA Sample Preparation kit (Illumina Inc., San Diego, CA, USA) and sequenced in a MiSeq platform (2 × 231 cycles paired-end run; Illumina).

### 2.3. Consensus SARS-CoV-2 and Intrahost Minor Variant Analysis

Generated reads were analyzed using Geneious R11 (Biomatters, Auckland, New Zealand). First, bases with quality below 30 Phreds and reads shorter than 60 bp were trimmed out using BBduk plugin. Reads were then assembled to the Wuhan-Hu-1 reference sequence genome (GenBank #MN908947) using three iterations, and the assemblies were visually inspected. Consensus sequences were retained when coverage was higher than 50% of the viral genome. Consensus sequences were extracted, and the different timepoints were compared within each patient. Single-nucleotide variant (SNV) positions identified between the longitudinal consensuses were evaluated for minor nucleotide frequency using the tool find variations/SNPs at Geneious R11 and independently using LoFreq v2.1.5, available on the Galaxy web platform [15,16]. Minor variants were defined as changes observed in sequences that differed from the consensus sequence and that were supported by both methodologies described above and with a minimum of 100× depth coverage and 2% frequency. Using those criteria, minor variants were considered non-spurious.

### 2.4. SARS-CoV-2 Classification and Case Definitions

To determine whether subjects have a sustained infection or were reinfected, a comprehensive phylogenetic analysis was performed. First, all generated sequences were classified with the NextClade web app [17]. Then a representative genomic dataset was assembled with sequences available on the GISAID EpiCoV database. The Audacity Instant app [18] was used to retrieve the 20 most similar sequences to each sequenced virus of this study. Sequences were aligned along the SARS-CoV-2 reference genome with the minimap2 [19] and gofasta [20] softwares, and untranslated flanking regions were trimmed from the alignment. A maximum likelihood tree was inferred with IQ-Tree 2 [21] with the GTR + F + I model, which presented the best fit according to ModelFinder [22]. To reconstruct mutations along branches of the tree, we performed an ancestral state reconstruction with TreeTime v.0.11.4 [23].

Through epidemiological and evolutionary analyses, we established parsimonious and objective criteria to classify cases as persistent or repeated infections (reinfections) based on phylogenetic reconstruction. These criteria are as follows: (1) In cases of confirmed persistence, sequences from both samples from the same subject cluster together in a clade with moderate-to-high statistical support (>70%) and display exclusive mutations (synapomorphies). (2) In cases of plausible persistence, sequences from both samples from the same subject cluster together, although the clade displays low statistical support and does not present synapomorphies. This category also includes cases where sequences display no phylogenetic resolution (both sequences cluster with zero support along a single polytomy). (3) In cases of confirmed reinfection, sequences from the same subject cluster with different reference genomes, composing distinct monophyletic clades. Both clades—and/or their exclusive containing clades—display moderate-to-high statistical support (>70%) and exhibit exclusive mutations (synapomorphies). (4) In cases of plausible reinfection, sequences from both samples from the same subject cluster with different reference genomes, composing distinct monophyletic clades. At least one of the clades displays moderate statistical support and exhibits exclusive mutations (synapomorphies). Additionally, sequences display incompatible sets of exclusive mutations.

### 2.5. Intrahost Evolutionary Rate

We were also prompted to investigate the intrahost virus evolutionary rates from the persistence cases. For each individual, we calculated the number of non-ambiguous exclusive mutations occurring along the branch connecting the latest sampled genome sequence to the ancestor of the clade that it composes with the first sampled sequence. We then obtained the numbers of mutations per site by dividing the number of exclusive mutations by the size of the SARS-CoV-2 reference genome (29,903 nucleotides). Finally, we divided these estimates by the time (in years) separating the dates of collection of the first and latest sample.

## 3. Results

**Figure 1:**
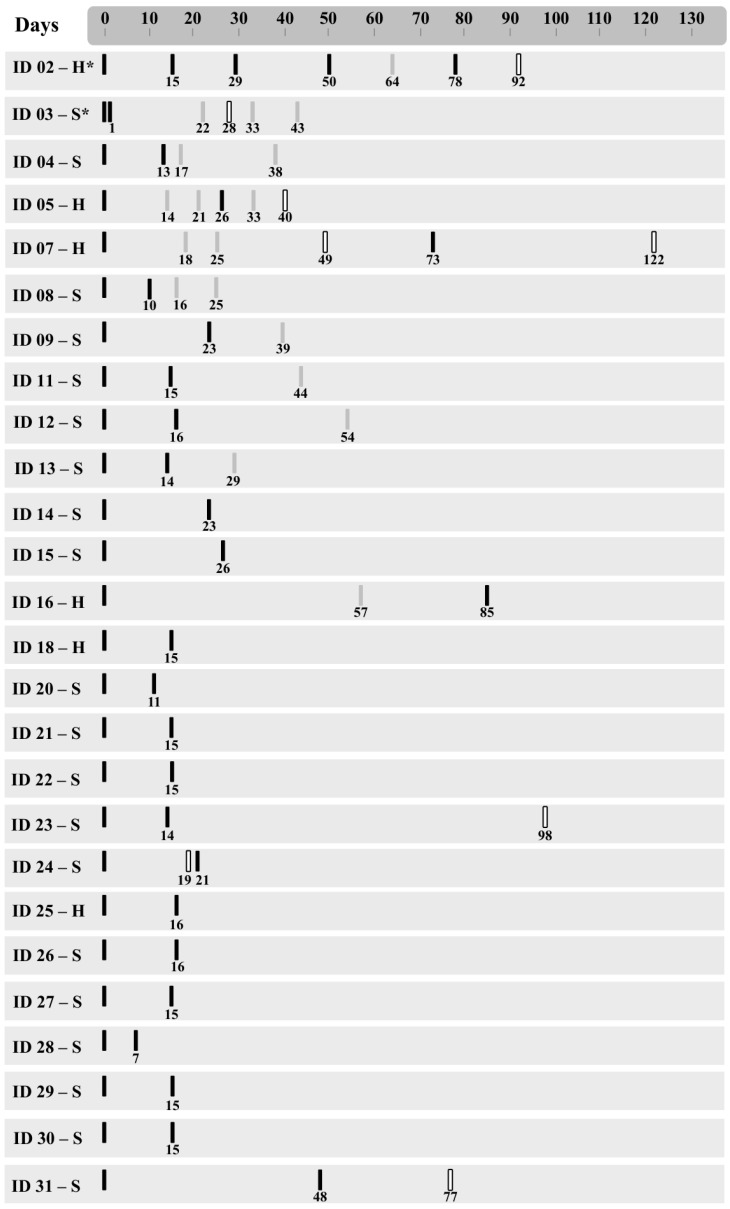
Timeline for SARS-CoV-2 RT-qPCR tests from patients included in the study. Black bars represent RT-qPCR-positive samples that were sequenced, gray bars show positive samples that were not sequenced, and white bars show RT-qPCR-negative samples. *H—Hematological cancer, S*—solid tumor.

A total of 61 SARS-CoV-2 RT-qPCR-positive samples derived from 28 different cancer patients were sequenced by ultra-deep sequencing. A mean of 549,310 reads was generated per sample, of which an average of 405,705 per sample remained after trimming for sequence quality (see Section 2). After assembly, four samples were excluded from analyses, because they did not present coverage higher than 50% of the viral genome. Of those, two samples corresponded to a third timepoint of two patients, and therefore these patients remained in the study with only two timepoints. The remaining two excluded samples corresponded to the second timepoint of two different patients who were excluded from the longitudinal analysis, as they remained with only one sampled timepoint. Most of the retained samples (79%) had more than 90% of the viral genome represented in the consensus sequence. Overall, 55 samples from 26 different patients remained for further analyses. Twenty-five patients had two samples analyzed, with the timespan between the samples ranging from 1 to 86 days. One patient had five samples analyzed, with a timespan between the first and the last sample of 78 days. The timeline with sample collection dates is shown in Figure 1, as are the cancer types (solid or hematological) that occurred in each patient.

Most of the sequences from samples collected in the beginning of the pandemic were classified as B1.1.33 (*n* = 38) and B.1.1.28 (*n* = 8). For the remaining nine, it was not possible to determine the genotype classification due to low coverage of genotype-specific signature positions.

### 3.1. Cases of SARS-CoV-2 Persistence

**Figure 2:**
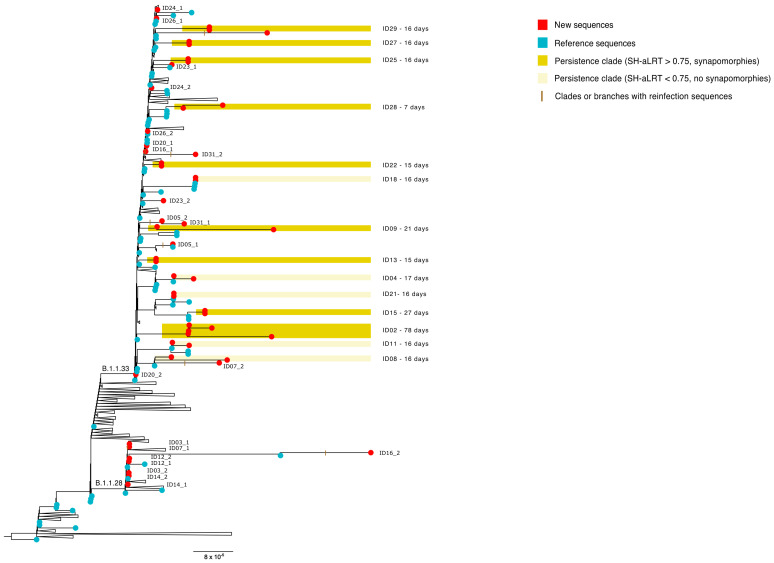
Maximum likelihood phylogenetic reconstruction of SARS-CoV-2 complete genomes sequenced in this study. Genomes generated in this study are represented by red circles and reference sequences by blue circles. Cases classified as confirmed persistent infections are highlighted in dark yellow. Plausible persistence cases, in which the sequences clustered with low statistical support, are shown in light yellow. Reinfection cases are marked with a brown bar on the corresponding clades or branches.

Confirmed SARS-CoV-2 persistence was observed in nine patients (ID02, ID09, ID13, ID15, ID22, ID25, ID27, ID28 and ID29), where sequences from different timepoints clustered with high statistical support in the phylogenetic reconstruction (Figure 2). In eight of these cases, two collection timepoints were sequenced, and the intervals between them ranged from 7 to 27 days. Interestingly, patient ID02 had five different samples analyzed, and the last one was collected 78 days after the first.

Plausible persistence criteria were observed in 12 cases. The sequences obtained for five of these patients (ID04, ID08, ID11, ID18 and ID21) clustered for each subject, with low statistical support in the phylogenetic tree (Figure 2). The remaining seven plausible persistence cases (ID03, ID12, ID14, ID20, ID23, ID24 and ID26) had sequences that displayed no phylogenetic resolution and exhibited polytomy in the phylogenetic reconstruction (Figure 2). The timespan between the two samples of each patient ranged from 1 to 26 days. It is worth noting that patient ID24 presented a negative RT-qPCR test among the two positive sequenced samples (Figure 1). This case was, therefore, suggestive of viral latency followed by reactivation, or a false negative RT-qPCR result of the intermediate swab collected from the patient.

All persistent cases with two or more SARS-CoV-2 genomes being successfully classified (*n* = 16) presented the same lineage at the different timepoints.

### 3.2. Cases of SARS-CoV-2 Reinfection

In five cases from 2020, the two sequences obtained from the same patient were distributed in different monophyletic clades (Figure 2). Three of them (ID05, ID07 and ID16) were classified as confirmed reinfection due to high statistical support in the cluster with different reference genomes and the presence of synapomorphies, while two (ID30 and ID31) were considered plausible reinfections, since sequences from both samples clustered with different reference genomes, but with moderate statistical support and incompatible sets of exclusive mutations. The time between the two collections in these cases ranged from 22 to 86 days in confirmed reinfections and from 14 to 48 days among the plausible reinfections. Among these reinfection cases, only ID16 had both sequences classified in SARS-CoV-2 lineages. The first genome was B.1.1.33, and the sequence from 86 days later was classified as B.1.1.28. Of note, only one case (ID07) in this category had a RT-qPCR-negative test between the two positive samples (Figure 1).

### 3.3. Persistence and Longitudinal Evolution

Variations in the consensus sequence over time were evaluated, comparing the viral genomes obtained for each persistent case. For six of the confirmed persistent (ID13, ID15, ID22, ID25, ID27 and ID29) and eight of the plausible persistence cases (ID3, ID12, ID14, ID18, ID20, ID21, ID24 and ID26), no nucleotide variations were observed between the two sequences obtained. Variations were found in seven cases (ID02, ID04, ID08, ID09, ID11, ID23 and ID28), as shown in Table 1. ID02 showed five positions that were maintained during the first four timepoints (timespan of 50 days) and changed in the last sample (78 days after the first sample). Furthermore, the genome of this last sample had a 15 nt deletion in the S gene that was not found in the two initial samples. The third and fourth collection timepoints were not analyzed for this deletion, as there was no coverage in this region. The fourth collection timepoint of ID02 also showed one exclusive single-nucleotide variation (SNV) when compared to the previous and following sequences. For ID04 and ID11, which had respectively 17 and 16 days between the samples, only one variation was observed. ID08 had three non-synonymous substitutions between the two sequences, collected 16 days apart. Regarding ID09, samples were collected with a timespan of 21 days, and three synonymous and one non-synonymous changes occurred between the two sequenced viruses. For ID23 and ID28, with sequences 17 and seven days apart, respectively, two SNVs were observed (Table 1).

The intrahost virus evolutionary rate was calculated based on the analysis of five individuals (ID02, ID04, ID08, ID09 and ID11) who presented mutations on the sequence of the second sample when compared to the ancestor and were estimated to be in the range of 7 × 10^−4^ − 2.3 × 10^−3^ substitutions per site per year.

### 3.4. Within-Host Viral Population

Positions that diverged among the longitudinal consensus of each case were evaluated for minor nucleotide frequency, and these results are presented in Table 2. For three persistent cases (ID04, ID08 and ID09), the positions of the major virus of the first timepoint were also observed as minor variants in the second timepoint. For these cases, data suggest that the second virus evolved from the first virus; however, the variation(s) that emerged did not get fixed between the two timepoints analyzed. The remaining persistent cases with longitudinal sequence variations represent infections with emergence of viral variations that got fixed over the time studied.

One case of plausible reinfection (ID31) showed variations from the second sample, which presented as minor variants in the first sample. This suggests a case of multiple infection in the first timepoint, followed by a clearance of the most prevalent virus identified at the first timepoint. The other reinfection cases showed no or very-low-frequency minor nucleotide variations, corroborating the hypothesis that the complete clearance of their first viral infection and the infection by a different virus occurred between the two timepoints analyzed. The last scenario does not exclude the occurrence of a multiple infection case before the clearance of the first infection.

## 4. Discussion

In this study, we report different longitudinal profiles of SARS-CoV-2 infection in cancer patients. Sustained viremia for up to 44 days, in some cases including virus evolution at 1–3 genomic positions, was the most frequent case. Infection by a new virus, simultaneously (coinfection) or after clearance of the first one (reinfection), was observed in seven cases.

It is well known that individuals who are immunocompromised have a higher risk of developing a persistent SARS-CoV-2 infection [24]. Under this scenario, several studies on longitudinal follow-up of SARS-CoV-2 infections have been reported [3,24]. Most of these focus only on specific individuals with suspected reinfection [24] and do not consider the possibility of a simultaneous infection by two strains at some point in the course of reinfection. In the results reported herein, we show one subject (ID9) carrying two different viral variants at the second collection point, one with SNV characteristics of the virus observed in the first timepoint, and the other with four divergent positions. Considering that all sequences from Rio de Janeiro state have an average of four SNVs between them, the number of changes found in ID9 in such a short period of time (23 days) suggests that this was a multiple infection case resulting from a novel viral infection combined with a previous active infection. In our case, the simultaneous infection occurred in a cancer patient who was treated for an inflammatory condition, with no development of respiratory symptoms. Patients’ comorbidities are associated with worse outcomes during SARS-CoV-2 infection [25] and can play a role in the susceptibility to multiple infections by two different strains, a scenario that is likely even more common than in immunocompetent subjects, something that has already been documented [26].

Other groups have reported reinfection cases at an interval of 4–20 weeks between the two positive swab collections [4,5,6,7,8,9,10]. Our cases are included in this range (3.5–12 weeks), except for ID8, where the interval between the two tests was only ten days. Specifically for this patient, the low coverage over some viral genomic fragments from the second collection did not allow us to analyze the minor variants. For this reason, we cannot rule out the possibility that the viral population observed at the first timepoint is still present in the second timepoint, characterizing a superinfection instead of reinfection. In a previous study, we reported a case where minor variants found in the first timepoint got fixed along with the infection and became the major population in a second timepoint, 106 days later [11]. This is clearly a case where minor variant analysis is an important tool to discriminate a multiple infection case from a *bona fide* reinfection.

COVID-19 reinfection usually occurs in individuals whose first episode was asymptomatic or with mild symptoms [27]. It has been reported that COVID-19 patients rapidly develop neutralizing antibodies, but asymptomatic patients have a weaker IgG response, and their antibodies decay more rapidly than in symptomatic or severe patients (ICU requirement) [28], and this can be a scenario where reinfection may occur [29,30,31]. Most of our patients were asymptomatic or developed mild symptoms, and one patient (ID2) had severe symptoms in the first episode and was asymptomatic in the second. We have not determined the antibody titers in our patients; therefore, we were not able to evaluate if antibody decay has played a role in reinfection. Another possibility would be that even if ID2 had produced long-lasting neutralizing antibodies, they would not or would less efficiently neutralize the second viral population observed in the course of infection, an occurrence that has also been shown in experimental animal models [31]. Specifically in this case, the second virus has a deletion of 15 bp and two amino acid changes (A22123C, K187N; T22912G, N450K) in the S protein, one of which is located at the receptor binding motif and may impact antibody recognition.

The within-host viral population analysis identified four different scenarios. The first one includes persistent cases where the major virus of the first timepoint was observed as a minor variant in the second timepoint. For these cases, data suggest that the second virus evolved from the first; however, the variation that emerged was not fixed between the two timepoints analyzed. This scenario also included patients with long periods of evolutionary stasis, supported by the absence of synonymous mutations due to little or no selective pressure [3].

The second scenario relates to the remaining persistent cases with longitudinal sequence variations that represent infections with emergence of viral variations that got fixed over the time studied. The third scenario suggests a case of multiple infection at the first timepoint, followed by a clearance of the most prevalent virus identified at the first timepoint. The variations identified at the second timepoint were present as minor variants in the first sample, which suggests a multiple infection case at the first timepoint. Finally, the last scenario includes reinfection cases with no or very-low-frequency minor nucleotide variations, corroborating the hypothesis that the complete clearance of their first viral infection and reinfection by a different virus occurred between the two timepoints analyzed. This last scenario does not exclude the occurrence of a multiple infection case before the clearance of the first infection.

Most patients studied herein only had two timepoints analyzed, even though some of them had more than two SARS-CoV-2-positive RT-qPCR results. This occurred because the methodology applied had limitations in terms of PCR amplification of specimens with Ct values greater than 35 [32]. We tried to improve this by designing a nested PCR rearranging the Arctic Network V3 multiplex primers into two PCR rounds; however, some samples remained unsuccessfully PCR-amplified. The presence of additional intermediate positive timepoints between samples limits the ability to reconstruct within-host evolutionary trajectories and may bias interpretation toward discrete categories rather than continuous dynamics. However, it is important to mention that we have classified cases in terms of persistence or reinfection based on phylogenetic reconstructions (by samples being clustered together with moderate-to-high statistical support or not), and not solely by the presence of specific nucleotide variants. Another limitation of the technique was the size of the fragments obtained. Both nested and direct PCR fragments have around 400 bp, and due to this limitation, it was not possible to confirm whether the polymorphisms found in a given sample belonged to the same viral variant (in-phase mutations).

For intrahost minor variant analysis, we applied a stringent minimum depth threshold of 100× coverage. This conservative criterion was intentionally chosen to minimize false-positive variant calls that could arise from sequencing artifacts or PCR-induced errors. As a consequence of this approach, several genomic positions with lower coverage are reported in Table 2 and were not analyzed for minor variant frequency. We acknowledge that by not analyzing those positions, we may have underestimated the minor variants detected, potentially limiting the ability to fully characterize multiple infections (as suggested for ID31) or identifying transient intrahost variants that emerged but did not become fixed between the timepoints studied (as observed for ID04, ID08, and ID09).

At the evolutionary level, a ~3-fold range in the estimated evolutionary rates among samples was observed. Although our estimates were consistent with previous studies [33,34], we emphasize that those rates were calculated from consensus-level differences between temporally separated samples and thus represent lower-bound estimates of within-host evolutionary change, without capturing transient variants, reversions, or heterogeneity in substitution rates over time. Another important limitation of our study lies in the resolution of phylogenetic analyses based on consensus sequences. Because longitudinal samples from the same patient were often separated by weeks and displayed few substitutions or incomplete genome coverage, phylogenetic reconstruction relied on a limited number of informative sites, potentially obscuring some cases of true persistence or reinfection. Future studies should incorporate denser longitudinal sampling and explicitly model within-host viral diversity, which would increase phylogenetic resolution and reduce ambiguity in distinguishing persistence from reinfection.

This is the first study that describes the use and importance of SARS-CoV-2 minor variants in distinguishing multiple infections from *bona fide* reinfections. To our knowledge, this is also the first time that not only SARS-CoV-2 reinfection, but also multiple infections and viral evolution over time have been reported in cancer patients. These patients are usually under treatments that negatively impact their immune system, and the effects of such adversity on the COVID-19 prognosis and on the occurrence of reinfections are unknown. Future studies investigating the SARS-CoV-2 intrahost diversity in immunodepressed patients may help improve our knowledge on how an impaired immune system reacts to the virus and the role of viral population dynamics in disease progression.

## Figures and Tables

**Table 1:** Single-nucleotide variations (SNVs) and indels found between SARS-CoV-2 genomes from the persistent cases.

Subject	SNV/Indel	AA Change
ID02	A9634T	ORF1:L3123F
	G10846A	ORF1:M3527I
	C18060T	None
	A22123C	S:K187N
	22286-22300del	S:del242-246
	T22912G	S:N450K
ID04	A13581G	None
ID08	G2036T	ORF1:A591S
	C20290T	ORF1:R6676W
	A24461T	S:S967C
ID09	T7657C	None
	T8217A	ORF1:V2651D
	C26340A	None
	G26925T	None
ID11	C20429T	ORF1:P6722L
ID23	T15179C	ORF1:V4972A
	A22779G	S:E406G
ID28	T7712C	ORF1:S2483P
	T22252A	None

Genome coordinates are based on SARS-CoV-2 Wuhan-Hu-1 reference sequence genome (GenBank acc. number MN908947).

**Table 2:** Minor nucleotide frequency distribution at positions with nucleotide variation between SARS-CoV-2 genomes from the first and the last samples of the studied cases.

Scenario	Subject	SNV/Indel	Minor Nucleotide Frequency		AA Change
			First Sample	Last Sample	
Confirmed Persistence	ID02	A9634T			ORF1:L3123F
		G10846A			ORF1:M3527I
		C18060T		C (4.04%)	
		A22123C	LC *	LC	S:K187N
		22286-22300del	LC	LC	S:del242-246
		T22912G			S:N450K
Plausible Persistence	ID04	A13581G		A (37.96%)	
Confirmed Reinfection	ID05	C6336T		C (2.29%)	
		T9474C		LC	
		T11471C			
Confirmed Reinfection	ID07	C186T			
		G970A			
		A2563C			
		T12053C			
		T25088G		LC	
		G25437T			
Plausible Persistence	ID08	G2036T	T (2.15%)	G (36.48%)	ORF1:A591S
		C20290T		LC	ORF1:R6676W
		A24461T		LC	S:S967C
Confirmed Persistence	ID09	T7657C		T (35.53%)	
		T8217A		LC	ORF1:V2651D
		C26340A		C (24.11%)	
		G26925T		LC	
Plausible Persistence	ID11	C20429T		LC	ORF1:P6722L
Confirmed Reinfection	ID16	C12053T		LC	ORF1:L3930F
		T13769A			ORF1:M4502K
		T18136G		LC	ORF1:F5958V
		A23772G		LC	S:D737G
		C23910T		LC	S:A783V
		T24016del		LC	S:Y818fs
		G25088T		LC	S:V1176F
Plausible Persistence	ID23	T15179C	LC		ORF1:V4972A
		A22779G	LC	LC	S:E406G
Confirmed Persistence	ID28	T7712C	LC		ORF1:S2483P
		T22252A	LC	LC	
Plausible Reinfection	ID30	G3210A	LC		ORF1:G982D
		G15211A	LC		ORF1:A4983T
		C18913T	LC		ORF1:R6217W
		T18929C	LC		ORF1:L6222P
Plausible Reinfection	ID31	C1385T	LC		ORF1:H374Y
		C2113T			
		T5065C	LC		
		T6336C	LC		ORF1:L2024S
		G18146A	A (39.17%)		ORF1:G5961E
		23288insG	insG (36.16%)		S: Y576fs restored

* LC: low coverage—positions with less than 100× depth coverage. Genome positions are based on SARS-CoV-2 Wuhan-Hu-1 reference sequence genome (GenBank number MN908947).

## Data Availability

SARS-CoV-2 consensus sequences generated in this study are available in GISAID under IDs EPI_ISL_20090097-146, EPI_ISL_20098365-67, and EPI_ISL_20096129, and sequencing data can be found in the Sequence Read Archive (SRA) under project number PRJNA657032.

## Supplementary Materials

The following supporting information can be downloaded at https://www.mdpi.com/article/10.3390/v18030393/s1: Table S1: List of primers used in the first round of the nested PCR for SARS-CoV-2 genome amplification.

## Abbreviations

The following abbreviations are used in this manuscript:

SARS-CoV-2	Severe acute respiratory syndrome coronavirus 2
SNV	Single-nucleotide variant
COVID-19	Coronavirus disease 2019
