# Supplementary material for: Disentangling SARS-CoV-2 Sustained Viremia Cases: Evolution, Persistence and Reinfection
Source: Viruses. 2026 Mar 21;18(3):393. doi: 10.3390/v18030393 (PMC13030517; doi:10.3390/v18030393)
Supplement: Supplementary file 1 [file viruses-18-00393-s001.zip › viruses-4120774-supplementary.pdf]

Supplementary Table S1. List of primers used in the first round of the nested PCR for SARS-CoV-2 genome amplification. All primers are from Artic Network version 3 protocol.

Pool	Primer name	Primer sequence
Pool 1	nCoV-2019_1_LEFT	ACCAACCAACTTTCGATCTCTTGT
	nCoV-2019_3_RIGHT	AAGGTGTCTGCAATTCATAGCTCT
	nCoV-2019_5_LEFT	TGGTGAAACTTCATGGCAGACG
	nCoV-2019_7_RIGHT	TGCACAGGTGACAATTTGTCCA
	nCoV-2019_9_LEFT	TCCCACAGAAGTGTTAACAGAGGA
	nCoV-2019_11_RIGHT	TCATCAGATTCAACTTGCATGGCA
	nCoV-2019_13_LEFT	TCGCACAAATGTCTACTTAGCTGT
	nCoV-2019_15_RIGHT	AACAGAAACTGTAGCTGGCACT
	nCoV-2019_17_LEFT	CTTCTTTCTTTGAGAGAAGTGAGGACT
	nCoV-2019_19_RIGHT	TGTCCAACTTAGGGTCAATTTCTGT
	nCoV-2019_21_LEFT	TGGCTATTGATTATAAACACTACACACCC
	nCoV-2019_23_RIGHT	ACCAGTACAGTAGGTTGCAATAGTG
	nCoV-2019_25_LEFT	GCAATTGTTTTTCAGCTATTTTGCAGT
	nCoV-2019_27_RIGHT	AATACAAGCACCAAGGTCACGG
	nCoV-2019_29_LEFT	ACTTGTGTTCCTTTTTGTTGCTGC
	nCoV-2019_31_RIGHT	ACAGAATAAACACCAGGTAAGAATGAGT
	nCoV-2019_33_LEFT	ACTTTTGAAGAAGCTGCGCTGT
	nCoV-2019_35_RIGHT	ACTTCATAGCCACAAGGTTAAAGTCA
	nCoV-2019_37_LEFT	ACACACCACTGGTTGTTACTCAC
	nCoV-2019_39_RIGHT	TGTAACTGGACACATTGAGCCC
	nCoV-2019_41_LEFT	GTTCCCTTCCATCATATGCAGCT
	nCoV-2019_43_RIGHT	AGCAGCATCTACAGCAAAAGCA
	nCoV-2019_45_LEFT	TACCTACAACTTGTGCTAATGACCC
	nCoV-2019_47_RIGHT	AATAACGGTCAAAGAGTTTTAACCTCTC
	nCoV-2019_49_LEFT	AGGAATTACTTGTGTATGCTGCTGA
	nCoV-2019_51_RIGHT	AGTGCATTAACATTGGCCGTGA
	nCoV-2019_53_LEFT	AGCAAAATGTTGGACTGAGACTGA
	nCoV-2019_55_RIGHT	GGTGTACTCTCCTATTTGTACTTTACTGT
	nCoV-2019_57_LEFT	ATTCTACACTCCAGGGACCACC
	nCoV-2019_59_RIGHT	AAGAGTCCTGTTACATTTTCAGCTTG
	nCoV-2019_61_LEFT	TGTTTATCACCCGCGAAGAAGC
	nCoV-2019_63_RIGHT	ACAAACTGCCACCATCACAACC

	nCoV-2019_65_LEFT	GCTGGCTTTAGCTTGTGGGTTT
	nCoV-2019_67_RIGHT	CAACCTTAGAAACTACAGATAAATCTTGGG
	nCoV-2019_69_LEFT	TGTCGCAAAATATACTCAACTGTGTCA
	nCoV-2019_71_RIGHT	TGGAAAAGAAAGGTAAGAACAAGTCCT
	nCoV-2019_73_LEFT	CAATTTTGTAATGATCCATTTTTGGGTGT
	nCoV-2019_75_RIGHT	ACCACCAACCTTAGAATCAAGATTGT
	nCoV-2019_77_LEFT	CCAGCAACTGTTTGTGGACCTA
	nCoV-2019_79_RIGHT	CATTTCATCTGTGAGCAAAGGTGG
	nCoV-2019_81_LEFT	GCACTTGGAAAACTTCAAGATGTGG
	nCoV-2019_83_RIGHT	TTTGACTCCTTTGAGCACTGGC
	nCoV-2019_85_LEFT	ACTAGCACTCTCCAAGGGTGTT
	nCoV-2019_87_RIGHT	ACTAGGTTCCATTGTTCAAGGAGC
	nCoV-2019_89_LEFT	GTACGCGTTCCATGTGGTCATT
	nCoV-2019_91_RIGHT	TTCAAGTGAGAACCAAAAGATAATAAGCA
	nCoV-2019_93_LEFT	TGAGGCTGGTTCTAAATCACCCA
	nCoV-2019_97_RIGHT	ACACACTGATTAAAGATTGCTATGTGAG
Pool 2	nCoV-2019_2_LEFT	CTGTTTTACAGGTTCGCGACGT
	nCoV-2019_4_RIGHT	CACAAGTAGTGGCACCTTCTTTAGT
	nCoV-2019_6_LEFT	GGTGTTGTTGGAGAAGGTTCCG
	nCoV-2019_8_RIGHT	GCTTCAACAGCTTCACTAGTAGGT
	nCoV-2019_10_LEFT	TGAGAAGTGCTCTGCCTATACAGT
	nCoV-2019_12_RIGHT	TTCACTCTTCATTTCCAAAAAGCTTGA
	nCoV-2019_14_LEFT	CATCCAGATTCTGCCACTCTTGT
	nCoV-2019_16_RIGHT	CACAACTTGCGTGTGGAGGTTA
	nCoV-2019_18_LEFT	TGGAAATACCCACAAGTTAATGGTTTAAC
	nCoV-2019_20_RIGHT	ACGTGGCTTTATTAGTTGCATTGTT
	nCoV-2019_22_LEFT	ACTACCGAAGTTGTAGGAGACATTATACT
	nCoV-2019_24_RIGHT	ACATTCTAACCATAGCTGAAATCGGG
	nCoV-2019_26_LEFT	TTGTGATACATTCTGTGCTGGTAGT
	nCoV-2019_28_RIGHT	TGTTTAGACATGACATGAACAGGTGT
	nCoV-2019_30_LEFT	GCACAACTAATGGTGACTTTTTGCA
	nCoV-2019_32_RIGHT	AGCACATCACTACGCAACTTTAGA
	nCoV-2019_34_LEFT	TCCCATCTGGTAAAGTTGAGGGT
	nCoV-2019_36_RIGHT	GAACAAAGACCATTGAGTACTCTGGA

	nCoV-2019_38_LEFT	ACTGTGTTATGTATGCATCAGCTGT
	nCoV-2019_40_RIGHT	CATGGCTGCATCACGGTCAAAT
	nCoV-2019_42_LEFT	TGCAAGAGATGGTTGTGTTCCC
	nCoV-2019_44_RIGHT	AACCTTTCCACATACCGCAGAC
	nCoV-2019_46_LEFT	TGTCGCTTCCAAGAAAAGGACG
	nCoV-2019_48_RIGHT	TAGATTACCAGAAGCAGCGTGC
	nCoV-2019_50_LEFT	GTTGATAAGTACTTTGATTGTTACGATGGT
	nCoV-2019_52_RIGHT	GTTGAGAGCAAAATTCATGAGGTCC
	nCoV-2019_54_LEFT	TGAGTTAACAGGACACATGTTAGACA
	nCoV-2019_56_RIGHT	ACACTATGCGAGCAGAAGGGTA
	nCoV-2019_58_LEFT	TGATTTGAGTGTTGTCAATGCCAGA
	nCoV-2019_60_RIGHT	GGTACCAACAGCTTCTCTAGTAGC
	nCoV-2019_62_LEFT	GGCACATGGCTTTGAGTTGACA
	nCoV-2019_64_RIGHT	AGTCTTGTAAAAGTGTTCCAGAGGT
	nCoV-2019_66_LEFT	GGGTGTGGACATTGCTGCTAAT
	nCoV-2019_68_RIGHT	CTCCTTTATCAGAACCAGCACCA
	nCoV-2019_70_LEFT	ACAAAAGAAAATGACTCTAAAGAGGGTTT
	nCoV-2019_72_RIGHT	ACTCTGAACTCACTTTCCATCCAAC
	nCoV-2019_74_LEFT	ACATCACTAGGTTTCAAACTTTACTTGC
	nCoV-2019_76_RIGHT	ACACCTGTGCCTGTTAAACCAT
	nCoV-2019_78_LEFT	CAACTTACTCCTACTTGGCGTGT
	nCoV-2019_80_RIGHT	TGGAGCTAAGTTGTTTAACAAGCG
	nCoV-2019_82_LEFT	GGGCTATCATCTTATGTCCTTCCCT
	nCoV-2019_84_RIGHT	AGGTGTGAGTAAACTGTTACAAACAAC
	nCoV-2019_86_LEFT	TCAGGTGATGGCACAACAAGTC
	nCoV-2019_88_RIGHT	TGGTCAGAATAGTGCCATGGAGT
	nCoV-2019_90_LEFT	ACACAGACCATTCCAGTAGCAGT
	nCoV-2019_92_RIGHT	AGGTTCCTGGCAATTAATTGTAAAAGG
	nCoV-2019_94_LEFT	GGCCCCAAGGTTTACCCAATAA
	nCoV-2019_98_RIGHT	TTCTCCTAAGAAGCTATTAAAATCACATGG
